# Genitourinary and Syphilitic Diseases

**Published:** 1903-09

**Authors:** Byron H. Daggett

**Affiliations:** Buffalo, N.Y.


### Genitourinary and Syphilitic Diseases.

CONDUCTED BY BYRON H. DAGGETT, M. D., Buffalo, N. Y.

### BACTERIURIA.

JANET (*Ann. des Malad. Organes Genito-urinaries,* No. 3, 1903). Urinary infection may be due to external and internal sources. Bacteriuria of external origin begins as an inflammation of the bladder mucous membrane, due to bacteria, usually the colon bacilli, ordinary bacilli or cocci. Prognosis is good in the absence of ureteritis and pelvitis. The treatment is bladder washings and instillation of silver salts. Bacteriuria of internal origin is obscure in origin and rebellious to treatment, since its cause is continuous. Bacteriuria of internal origin may be due to the proximity of an infected organ, or the infection may be delivered by means of the circulation. It is generally due to cocci, sometimes to the colon bacillus.

Bacteriuria from indirect contact or proximity, follows prostatic infection or a vesiculitis, which may repeatedly infect the bladder. It is needful to know whether or not prostatitis be the cause or effect of bacteriuria, since microbes eliminated through the urine may cause urethritis, if these bacteria be infections to the urethral membrane.

To determine whether bacteriuria is due to infection of the lower urinary tract or the kidneys, wash the bladder, and the urine will show but few or no bacteria; if it be renal, the urine will still show abundant germs.

The treatment of vesical or prostatic bacteriuria is local, and consists of lavage, massage, instillations and disinfection. Urotropin is especially valuable, together with benzoate of soda, if the urine be alkaline.

Bacteriuria of internal origin may arise from the rectum, intestines and the genital and urinary organs, and is more common than is usually believed to be. Bacteriuria through the blood is typified by the urinary infection of typhoid fever. Diagnosis of this form is very difficult. It is characterised by the evolution of a series of paroxysms, accompanied by fever.

Membranous enteritis may cause colon bacillus to appear in the urine. The persistent cystitis of constipated women is undoubtedly due to the colon bacillus. The route taken by the bacilli is unknown. Urinary remedies are useless. Milk diet, purgation, normal salt solution in enema are recommended. Treatment to be kept up for three months. Urotropin is the best internal remedy—twenty grain doses three times daily. Keep urine acid. Patients should be tested for idiosyncrasy before giving large doses of urotropin.

---

THE MECHANICS OF MERCURY IN THE TREATMENT OF SYPHILIS.

THOMAS W. MERRILL (*The Atlanta Journal-Record of Medicine,* March, 1903,) says the use of mercury in the treatment of syphilis is very ancient, the first were the Chinese ages ago. It is known as a specific and only a charlatan or ignoramous would deny its preëminence in syphilitic therapy. Still there is a wide difference in the methods of its use and the choice of the form to be used.

The pathology of this disease is exceedingly simple and consists solely of "an active proliferation of the normal germinal cells of the parts." The virus of syphilis produces a stimulating effect on all the cells of the economy, some parts being influenced more than others and such parts proliferate very rapidly. The lymph glands are first attacked and enlarge. It travels the lymph channels on through the thoracic duct the subclavian vein and, becoming absorbed, affects all the tissues of the body. There is no inflammation, the swelling is due to the increased number of cells and not to their swelling. The glands are hard and dense, not much enlarged and remain enlarged after the disappearance of the chancre. The skin eruption is due to the proliferation of the epithelial cells.

Proliferating cells block the lymphatic channels, disturbing their function, the proliferation goes on and the masses of cells not being carried off make their appearance as gumma in the tertiary stage.

Syphilis is as much a self-limiting disease as measles and mercury has no antitoxic action upon syphilitic virus. Mercury produces its results mechanically, by clearing in its passage the lymph channels. Iodide of potassium increases the stimulation caused by the virus and does harm in the active stages of this disease, while mercury clears the channels and maintains their function. The globules of mercury in their passage through the lymph channels carry forward the detritus and the function of the channel is preserved. The finer the subdivision of the mercury the greater is its action and the more thorough are its results. Under the microscope the globules are found crowding the finest channels. The finer divided forms are more absorbable, hence preferable.

The protoiodide is the most used and the most useless. Dr. Julien says that the protoiodide is the father of stomatitis. This iodide will produce an eruption like syphilis. Its action is uncertain—a reaction has set in and iodine compounds are under the ban. The blue ointment by inunction is the best. More mercury can be given in the form of calomel and blue powder than in any other way, hence they are the best. Clinical observations have demonstrated that the finely divided milder forms of mercury are the best. Where the syphilis is dead iodide may be used to clear up the debris.

